# Pregnancy outcomes in women with pemphigus exposed to rituximab before or during pregnancy

**DOI:** 10.1097/JW9.0000000000000038

**Published:** 2022-07-12

**Authors:** Azin Dehghanimahmoudabadi, Nika Kianfar, Marwa Akhdar, Shayan Dasdar, Kamran Balighi, Hamidreza Mahmoudi, Maryam Daneshpazhooh

**Affiliations:** a Autoimmune Bullous Diseases Research Center, Tehran University of Medical Sciences, Tehran, Iran.

**Keywords:** Autoimmune, pemphigus, pregnancy, rituximab, women

## Abstract

Rituximab (RTX) is an effective treatment for pemphigus; however, the drug labeling recommends not to use RTX within 1 year before conception.

**Objectives:**

To report pregnancy outcomes of patients with pemphigus who were treated with RTX before or during pregnancy.

**Methods:**

We identified 19 pregnancies with RTX exposure before or during pregnancy. All had previously been advised not to get pregnant within 1 year of RTX administration. The cases were categorized into 3 groups of exposure of within 6 months (group A), between 6 and 12 months (group B), and longer than 12 months of conception (group C). The pregnancy outcomes of different RTX exposure intervals were compared.

**Results:**

Group A included 9 pregnancies, of which 3 had received RTX accidentally after conception. Group B and C included 4 and 6 pregnancies, respectively. There was no significant difference between the groups regarding pregnancy outcomes. Overall, there were 17 live births, 1 spontaneous abortion, and 1 termination. Of the live births, 3 preterm deliveries and 4 low-birth-weight neonates were noted. Moreover, 1 neonate was hospitalized due to early-onset neonatal sepsis, and 1 had hydronephrosis. Disease flare-up occurred in 5 patients during pregnancy (4 minor and 1 major relapses) and in 5 patients after delivery (3 minor and 2 major relapses).

**Conclusions:**

Except for 1 case of neonatal sepsis which survived following medical treatment, no serious relevant adverse pregnancy outcome that could be attributed to RTX exposure before and during early pregnancy in women with pemphigus was detected. Nevertheless, RTX should not be administered within 1 year before planned pregnancy, as not enough data is available yet.

**What is known about this subject in regard to women and their families?**

- Women with pemphigus are at risk of disease worsening during and after pregnancy.
- Disease management in this period is challenging as newborns’ health may be affected.
- According to drug labeling, rituximab is not recommended within 1 year of pregnancy.

**What is new from this article as messages for women and their families?**

- Except 1 case with neonatal sepsis, no serious relevant adverse pregnancy outcomes occurred by rituximab within 1 year of accidental pregnancy.
- Good control of disease during and after pregnancy was achieved in this series.

## Introduction

Pemphigus is a group of severe and life-threatening autoimmune diseases characterized by blisters and erosions on the skin and mucosa. The disease is mediated by autoantibodies mainly against desmoglein (Dsg) 1 and 3, responsible for intercellular adhesion.^1-3^ Patients with pemphigus may experience disease flare-up from alterations in immune responses throughout pregnancy.^4^ Moreover, transplacental transfer of maternal antibodies may cause neonatal pemphigus.^5^

For women of child-bearing age, despite the contraceptive counseling, treatment strategies should be chosen with consideration of probable pregnancy. Given the potential risk of immunosuppressive medications for the fetus, management of pemphigus before and during pregnancy is challenging.^6^

Rituximab (RTX) is a chimeric monoclonal antibody directed against CD20-expressing B-lymphocytes.^3,7^ Recently, RTX received FDA approval for its promising therapeutic effects in adult patients with moderate to severe pemphigus. The drug has a long half-life averaging between 8 and 22 days and leads to prolonged depletion of B cells for up to 12 months after infusion.^3^

RTX, as a monoclonal IgG1 antibody, is transferred across the placenta through Fc receptors time-dependently. In other words, the passage during the first trimester is insignificant, whereas the maximum transfer occurs during the last 4 weeks of pregnancy.^8^ Therefore, despite its safety in animal studies, product labeling recommends discontinuation of RTX 12 months before conception.^9^ However, some pregnancies may occur within this period despite physicians’ warnings. Data regarding pregnancy outcomes after RTX exposure in pemphigus are limited to a few case reports. Herein, we aimed to assess the pregnancy outcomes in a series of 19 women with pemphigus treated with RTX before or during pregnancy.

## Methods

### Study design and patients

This cross-sectional case series was performed at the dermatology referral center of Razi hospital, Tehran University of Medical Sciences, Tehran, Iran. We searched electronic databases to identify women with a confirmed diagnosis of pemphigus who received RTX infusions between June 2011 and January 2020. The diagnosis of pemphigus was established according to the clinical, histopathological, direct immunofluorescence, or anti-Dsg 3/1. Subjects with a reported pregnancy exposed to RTX any time before or during pregnancy were screened for inclusion. The study protocol was reviewed and approved by the Tehran University of Medical Sciences Research Ethics Committee (IR.TUMS.MEDICINE.REC.1396.3002).

### Clinical assessments and treatment approach

Nineteen pregnancies from 16 women with pemphigus who had exposure to RTX before conception through to delivery were identified. The indication for RTX administration was in accordance with Iranian guidelines.^10^ Patients’ data regarding diagnosis, age at onset, number of RTX courses, dates of RTX administration, age at pregnancy, date of pregnancy, time interval between RTX administration and conception, number of pregnancies after RTX administration, dose of oral corticosteroid given during pregnancy, relapse of pemphigus during or after pregnancy, and other concomitant drugs were collected. Patients were stratified according to the time interval between RTX and conception into 3 groups, group A: RTX during pregnancy or pregnancy interval until conception equal or less than 6 months; group B: more than 6 months until 12 months; group C: more than 12 months. Group C served as the control group.

The following data regarding pregnancy outcomes were also recorded: live birth, gestational age (GA) at labor (preterm labor: <37 weeks of GA), birth weight (low-birth-weight: <2500 g), presence of major anomaly, early-onset neonatal sepsis, spontaneous abortion (before 20 weeks of GA), stillbirth (after 20 weeks of GA), and termination.

### Statistical analysis

Absolute numbers with frequency were used for reporting the categorical data, and mean ± SD with range were used for the continuous data. The Fisher-Freeman-Halton exact, Kruskal-Wallis, and Mood’s median tests were used to compare the differences between the groups. *P* values <0.05 were considered statistically significant. All analyses were conducted using SPSS 24.0 (IBM Corp, NY).

## Results

### Characteristics of patients treated with RTX

Fourteen women were diagnosed with mucocutaneous pemphigus vulgaris (PV) (88%), one with mucosal PV (6%), and one with pemphigus foliaceus (PF) (6%). The mean age of the patients was 28.6 ± 5.5 years (20–40) at pemphigus diagnosis and 32.3 ± 5.4 years (23–44) at conception. Based on the timing of last exposure to RTX, 9 pregnancies were assigned to group A, 4 pregnancies to group B, and 6 pregnancies to group C. Three women had got pregnant twice; all with an additional RTX cycle between 2 pregnancies. Both pregnancies of 2 women belonged to group A and the other woman’s pregnancy was once in group B and then in group C. In group A, 3 women received RTX inadvertently during the first trimester of pregnancy. No exposure was recorded in the second or third trimester. Maternal age, RTX cycles before conception, cumulative dose of last RTX cycle, and prednisolone consumption during pregnancy were not statistically different between groups (Table 1).

**Table 1:** Baseline characteristics of 19 pregnancies in women with pemphigus ever treated with rituximab

Characteristics	Exposed to RTX before pregnancy				
	Total number	≤6 mo (group A)	6–12 mo (group B)	>12 mo (group C)	*P*
Pregnancies (women), n	19 (16^a^)	9 (7)	4 (4)	6 (6)	
Interval between last RTX infusion and conception, mo, mean ± SD^b^	10.8 ± 9.8	2.2 ± 1.1	8.8 ± 0.9	20.6 ± 9.0	
Age at disease diagnosis, y, mean ± SD	28.6 ± 5.5	28.7 ± 6.1	29.2 ± 7.	27.1 ± 3.9	0.619^c^
Age at conception, y, mean ± SD	32.3 ± 5.4	31.5 ± 5.4	32.5 ± 8.0	33.1 ± 4.7	0.751^c^
RTX cycles before conception, median (range)	1	2 (1-8)	1 (1-2)	1 (1-2)	0.512^d^
Cumulative dose of last RTX cycle, mg, mean ± SD	1841 ± 374	1666 ± 500	2000 ± 0	2000 ± 0	0.153^c^
Patients with more than minimal therapy (>10 mg prednisolone) during pregnancy, n (mean, mo)	8 (5)	5 (6)	0 (-)	3 (3)	0.155^e^

a One woman was same in group B and C.

b Those with RTX exposure during pregnancy were not included for interval calculation.

c Based on the Kruska-Wallis test.

d Based on the Median test.

e Fisher Freeman Halton Exact.

RTX, rituximab.

### Pregnancy outcomes

**Fig. 1.**
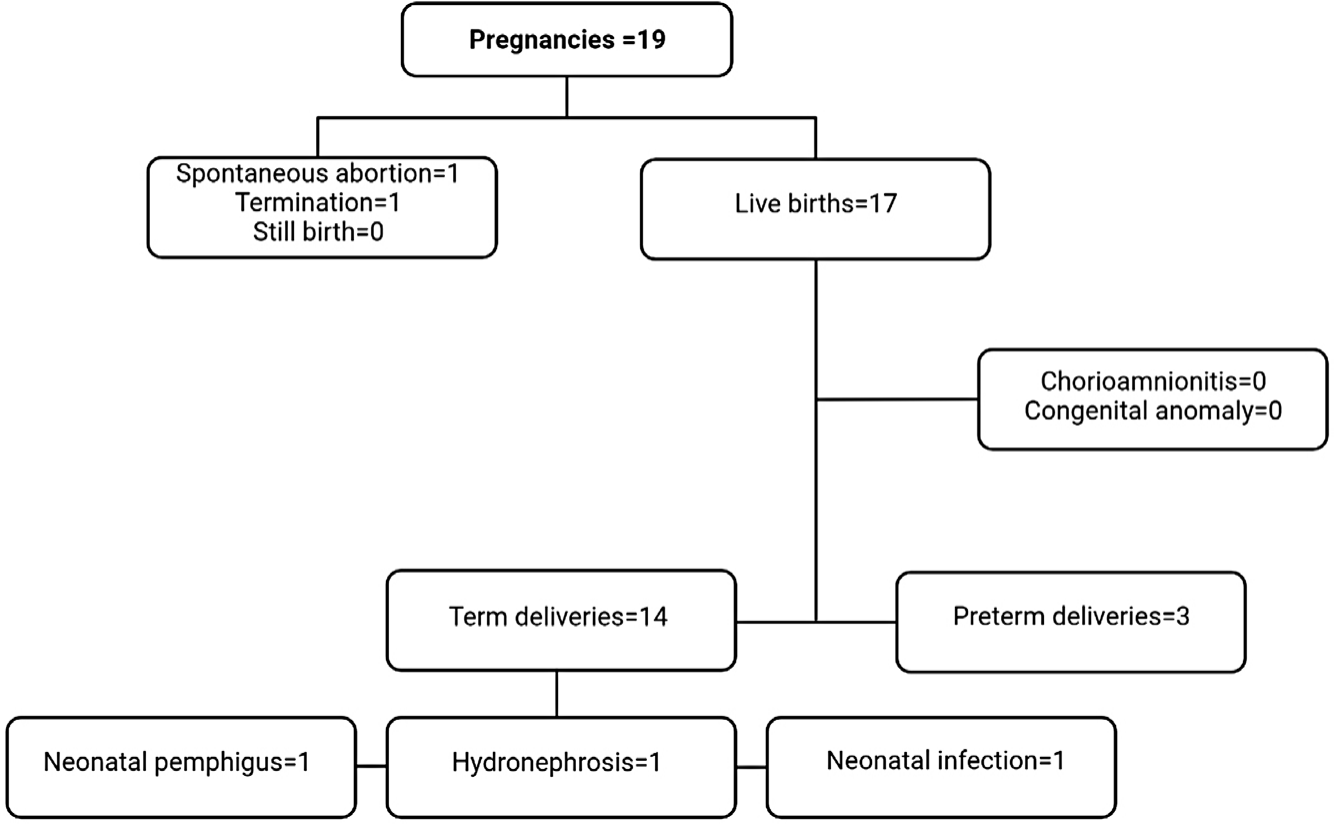
Pregnancy outcomes following rituximab exposure in patients with pemphigus.

Of 19 pregnancies, 17 (89%) resulted in live births, one in termination (5%), and one in spontaneous abortion (5%). Three preterm deliveries (17%) and 4 low-birth-weight neonates (23%) were reported among live births. There was no case of maternal death, neonatal death, or chorioamnionitis in this series (Figure 1). The mean birth weight in term infants was 3017 ± 419 g, and nonsignificantly lower in newborns of group A (2941 ± 409 g) compared with the other 2 groups (group B: 3363 ± 349 g and group C: 2980 ± 370 g). None of the living neonates was identified with a major anomaly, although 1 experienced an episode of early-onset neonatal sepsis.

Group A included 9 pregnancies in 7 women who received RTX during pregnancy or within 6 months before conception. Regarding pregnancy outcomes of group A, there were 1 medical termination, 2 preterm delivery, and 3 low-birth-weight neonates. The termination was due to a screen-positive result for Down syndrome in a 35-year-old woman who inadvertently had RTX infusion at 1 month of gestation. Notably, this woman was receiving mycophenolate mofetil and cotrimoxazole up to pregnancy detection. One woman who accidentally received RTX 2 months after conception had a neonate with left kidney hydronephrosis. The boy suffered from recurrent urinary tract infections, which resolved without any surgical intervention within his first year of life.

Group B included 4 pregnancies who had received their last infusion between 6 and 12 months before pregnancy. Regarding the adverse pregnancy outcomes, 1 spontaneous abortion and 1 early-onset neonatal sepsis were recorded in this group. First trimester spontaneous abortion (8-week GA) for an unknown reason occurred to a 27-year-old woman who conceived after 9 months of RTX exposure. Notably, the woman was under methotrexate treatment that was stopped 2 months before conception. One case of early neonatal sepsis happened to a 32-year-old woman who conceived 8 months after RTX exposure. The full-term boy, after birth, was hospitalized for 10 days and treated with appropriate antibiotics; he survived with no long-term problem.

Group C included 6 pregnancies who had RTX exposure for longer than 12 months before pregnancy. Among the offspring, 1 preterm delivery with low-birth-weight and 1 neonatal pemphigus were detected. A 40-year-old woman who had relapsed at 7 months of gestation gave birth to a boy with neonatal pemphigus whose lesions resolved 2 weeks after topical steroid therapy. The outcome of pregnancy in each group is depicted in Table 2.

**Table 2:** Pregnancy outcomes following rituximab exposure in patients with pemphigus

Pregnancy outcome	Exposed to RTX before pregnancy				*P*
	Total number	≤6 mo (group A) (n = 9)	6–12 mo (group B) (n = 4)	>12 mo (group C) (n = 6)	
Live birth, n (%)	17 (89%)	8 (88%)	3 (75%)	6 (100%)	0.684^a^
Termination, n (%)	1 (5%)	1 (11%)	0	0	1.000^a^
Spontaneous abortion, n (%)	1 (5%)	0	1 (25%)	0	0.211^a^
Stillbirth, n (%)	0	0	0	0	-
Preterm delivery, n (%)	3 (17%)	2 (25%)	0	1 (16%)	1.000^a^
Low-birth-weight, n (%)	4 (23%)	3 (37%)	0	1 (16%)	0.461^a^
Early-onset neonatal sepsis, n (%)	1 (6%)	0	1 (25%)	0	0.176^a^
Neonatal pemphigus, n (%)	1 (6%)	0	0	1 (16%)	0.526^a^
Infants with major congenital anomaly, n (%)	0	0	0	0	-
Birth weight of term infants, g, mean ± SD	3017 ± 419	2941 ± 409	3363 ± 349	2980 ± 370	0.247^b^
Birth weight of all infants, g, mean ± SD	2807 ± 666	2567 ± 794	3363 ± 349	2850 ± 459	0.149^b^

a Based on the Fisher-Freeman-Halton exact test.

b Based on the Kruskal-Wallis test.

RTX, rituximab.

### Relapse during and after pregnancy

Five (26%) relapses occurred during pregnancy, 2 in group A (22.2%) and 3 in group C (50%). In group A, there was 1 minor relapse treated with minimal oral corticosteroid therapy (prednisolone ≤10 mg/daily) up to the end of pregnancy and 1 major relapse managed with 6 cycles of IVIg infusion. All pregnancy relapses in group C were minor and managed with an increased dose of oral corticosteroids (maximum 5 mg/daily increase). Furthermore, 5 relapses (26%) within 1.3 ± 1.2 (0–3) months postpartum were documented, 2 of which belonged to group B (50%), and 3 belonged to group C (50%). No postpartum relapse occurred in women of group A.

The woman with spontaneous abortion in group B had a postpartum disease flare-up after 2 weeks. The other relapse in this group occurred 1 month after delivery. Both patients were administered with an additional cycle of RTX (4 weekly infusions) due to the severity of the relapses. In group C, 3 women had a reactivation of their disease after delivery. Of them, 2 were managed with minimal oral corticosteroid therapy, and one with adjuvant mycophenolate mofetil therapy (The detailed information of each woman regarding the disease, pregnancy, and treatments are shown in Supplementary Table S1, http://links.lww.com/IJWD/A8).

## Discussion

Most studies regarding pregnancy exposure to RTX are about women with malignancies, as well as neurologic and rheumatologic disorders.^11-17^ Herein, we reported the outcomes of 19 pregnancies in women with pemphigus who received RTX before or during pregnancy.

Of the all exposed pregnancies, 89% ended in live births with 17% preterm deliveries, which was consistent with the general population rate.^18^ In this series, no infant with major congenital anomaly nor stillbirth was reported although one was affected with neonatal pemphigus. In a similar report from our center before RTX being available, the rate of live births and preterm deliveries in 52 pregnant patients with pemphigus were 89% and 13%, respectively. There were also 4 cases of stillbirth, meningocele, neonatal pemphigus and intrauterine growth retardation. Thus, it seems that use of RTX in the current study has not been associated with increased pregnancy complications.^19^

We also found no serious adverse pregnancy outcomes occurred by RTX infusion within 6 months or between 6 and 12 months of accidental pregnancy. The risk of low-birth-weight was not significantly different between particular exposure intervals. However, more low-birth-weight neonates were recorded when RTX was administered within 6 months of conception, which can be attributed to the underlying disease or higher doses of prednisolone.

The largest report regarding RTX exposure in other chronic diseases relates to the study derived from the global drug safety database.^8^ They evaluated 153 pregnancies with known outcomes who had preconception or antepartum RTX exposure. In that study, the rate of live births was 79%, preterm delivery was 19%, and the congenital anomaly was 2%.

In a recent cohort of patients with neuroimmunological disorders, the pregnancy outcomes following anti-CD20 therapies were investigated.^11^ Evaluating the data of 68 pregnancies demonstrated an overall live birth of 88% and preterm birth of 15%. They found that patients with RTX exposure during pregnancy had a higher risk for preterm delivery and low-birth-weight, which was ascribed to the recent disease activity.

Neonatal infection subsequent to the probable hypogammaglobulinemia is one of the main concerns regarding RTX therapy around pregnancy.^20^ In a review of 22 studies on RTX therapy in pregnant women, B-cell depletion was identified in 9 of 23 (39%) pregnancies that all reached the normal level 6 months afterward.^14^ Neonatal sepsis is the only adverse outcome relevant to the pharmacokinetics of the RTX. Although one case of neonatal sepsis occurred in this study, it corresponded to the estimate of neonates at birth (the national prevalence of sepsis in our country is estimated to be 15.98%).^21^ It is worthy to note that most studies indicated no increased risk of infection in RTX exposed pregnancies^11,13-15^; nevertheless, close monitoring of neonates is crucial to detect the potential infection promptly.

Data concerning pregnancy outcomes in women with pemphigus who conceive after RTX infusion are very limited. Of note, in the present study, 3 patients received RTX during pregnancy. One terminated owing to a high probability of Down syndrome, one resulted in a term baby with hydronephrosis, and one resulted in a healthy neonate. Trisomy 21 is due to maternal nondisjunction, but our case received RTX 1 month after conception. So, it is hard to consider this phenomenon as an RTX complication; and it could have pertained to the mycophenolate mofetil or cotrimoxazole coexposure in that pregnancy.^25^ Likewise, the metanephros formation begins during the fifth week of gestation, and there is very little chance that RTX has affected this process.

Patients with pemphigus are susceptible to disease exacerbation during and after pregnancy; thus, its management is a crucial issue. Treatment with new modalities like RTX will delay the risk of disease flare-up.^4^ Our patients demonstrated remarkably fewer relapses during pregnancy and postpartum compared with the course of pemphigus in pregnancy (26% vs 54% during pregnancy and 26% vs 44% postpartum).^19^

RTX received the FDA approval for adult patients with pemphigus; however, according to the drug leaflet it is contraindicated in pregnant women, within 1 year of conception and during lactation, and is not yet approved for childhood pemphigus. Case series are useful means of reporting the outcomes of RTX exposure, and several studies indicated no increased risk of harmful outcomes to the mentioned groups.^5,26-28^ Based on the evidence above, no major safety signal was detected regarding RTX exposure around pregnancy. However, scarce available data preclude recommending RTX administration for control of pemphigus during pregnancy especially considering the risk of neonatal hypogammaglobulinemia. It can just be implied that there is not much concern for adverse outcomes in the case of inadvertent exposure.

Aside from the small number of samples due to the nature of this study, the main limitation was the lack of B-cell measurements. Moreover, the short follow-up of the exposed neonates makes it difficult to draw a definite conclusion about the drug’s complications. Notwithstanding, our study constituted the largest case series concerning prenatal exposure to RTX in patients with pemphigus. Larger studies evaluating pregnancy outcomes following RTX exposure and measurement of neonatal B cells are needed to underscore our findings.

## Conclusions

Our preliminary data demonstrated that inadvertent usage of RTX before and during the early pregnancy was not accompanied by any major relevant complications in pemphigus patients and their neonates, except for one case of neonatal sepsis. However, we still recommend that women of child-bearing age avoid pregnancy for 1 year after RTX.

## Author contributions

K.B., H.M., M.D.: Conception of the work.A.D.: Data collection.A.D., N.K., S.D.: Data analysis.N.K., M.A., S.D.: Drafting the article.N.K., S.D., H.M., M.D.: Critical revision.A.D., N.K., M.A., S.D., K.B., H.M., M.D.: Final approval of the version to be published.

## Conflicts of interest

None.

## Funding

None.

## Study approval

This study was approved by IR.TUMS.MEDICINE.REC.1396.3002.

## Supplementary materials

Supplementary material associated with this article can be found at http://links.lww.com/IJWD/A8.

## Supplementary Material


