# Supplementary material for: Pregnancy outcomes in women with pemphigus exposed to rituximab before or during pregnancy
Source: Int J Womens Dermatol. 2022 Jul 12;8(3):e038. doi: 10.1097/JW9.0000000000000038 (PMC9276145; doi:10.1097/JW9.0000000000000038)
Supplement: Supplementary file 1 [file jw9-8-e038-s001.pdf]

Supplementary Table 1 Data summary of women with pemphigus exposed to rituximab before or during pregnancy

Pregnancy's number	Diagnosis	Age at pregnancy (years)	No. of RTX cycles	Last RTX infusion dose (mg)	Duration between RTX infusion and conception	Months with more than minimal therapy (>10 mg prednisolone) during pregnancy ~~Average PSL dose during pregnancy (mg/day)~~	Recent immunomodulatory/other medications~~medication before conception~~	Relapses during pregnancy (month GA)/ treatment	Relapses after pregnancy (month postpartum)/ treatment	Preterm delivery	Live birth	Sex	Weight (gr)	Adverse pregnancy outcomes
1	MCPV	40	4	2000	2 months GA	~~5~~1	PSL/-	-	-	-	+	M	3150	hydronephrosis
2	MCPV	35	1	1000	1 month GA	~~12.5~~6	PSL, Mycophenolate mofetil (until conception)/ Cotrimoxazole (one week at the early conception)	-	-	-	-	-	-	Termination due to high probability of Down syndrome
3	MCPV	27	2	2000	0	~~15~~9	PSL, Methotrexate (until 1 month of conception)/ Acyclovir	-	-	-	+	F	3500	
4	MCPV	27	2	1000	1 week	~~10~~8	PSL/-	5, 8/PSL	-	+	+	F	2400	
5	MCPV	29	2	1000	2 months	~~20~~7	PSL/-	4/IVIg	-	+	+	M	890	
6	MCPV	32	1	2000	2 months	5	PSL/ Isoniazid (until two months after conception)	-	-	-	+	M	3150	
7	MCPV	23	1	2000	3 months	6.4	PSL/-	-	-	-	+	F	2450	
8	MCPV	36	1	2000	3 months	1.5	PSL/ Cotrimoxazole (one week at the early conception)	-	-	-	+	M	2500	
9	MCPV	35	8	2000	3 months	0	-/-	-	-	-	+	M	2500	
10	MCPV	32	1	2000	8 months	0	PSL/-	-	1/RTX	-	+	M	3760	Early-onset neonatal sepsis
11	MCPV	44	2	2000	8 months	0	-/Levothyroxine	-	-	-	+	M	3100	
12	MCPV	27	1	2000	9 months	5	PSL, Methotrexate (until 2 months of conception)/ Levothyroxine	-	0.5/RTX	-	-	-	-	Spontaneous abortion (8 week GA)
13	MCPV	27	1	2000	10 months	5.6	PSL/-	-	-	-	+	M	3230	
14	MCPV	30	2	2000	13 months	0.6	PSL/ Levothyroxine	-	-	-	+	F	3000	

15	MCPV	30	2	2000	13 months	~~7.9~~5	PSL/Insulin	-	-	-	+	F	2900	
16	MCPV	28	1	2000	16 months	~~6.1~~2	PSL/-	6/PSL	0/PSL	+	+	F	2200	
17	MPV	37	1	2000	18 months	2.5	PSL/-	-	-	-	+	F	2700	
18	MCPV	40	1	2000	31 months	~~6~~2	PSL/-	7/PSL	2/PSL, Mycophenolate mofetil	-	+	M	2700	Neonatal pemphigus
19	PF	34	1	2000	33 months	0	-/Aspirin	2/PSL	3/PSL	-	+	M	3600	

Abbreviations: PF, pemphigus foliaceus; MPV, mucosal pemphigus vulgaris; PV, pemphigus vulgaris; PSL, prednisolone; GA, gestational age; RTX, rituximab; IVIg, intravenous immunoglobulin
